# Downregulation of Homer1b/c in SOD1 G93A Models of ALS: A Novel Mechanism of Neuroprotective Effect of Lithium and Valproic Acid

**DOI:** 10.3390/ijms17122129

**Published:** 2016-12-17

**Authors:** Hai-Zhi Jiang, Shu-Yu Wang, Xiang Yin, Hong-Quan Jiang, Xu-Dong Wang, Jing Wang, Tian-Hang Wang, Yan Qi, Yue-Qing Yang, Ying Wang, Chun-Ting Zhang, Hong-Lin Feng

**Affiliations:** Department of Neurology, the First Affiliated Hospital of Harbin Medical University, Harbin 150001, China; jianghz@hrbmu.edu.cn (H.-Z.J.); WangS319@hotmail.com (S.-Y.W.); YinXiang15@hotmail.com (X.Y.); jianghongquan321@hotmail.com (H.-Q.J.); wangxudonghlj@hotmail.com (X.-D.W.); hebgdwangjing@163.com (J.W.); libu979@126.com (T.-H.W.); qiswallow21@sohu.com (Y.Q.); yangyueqing714@sina.com (Y.-Q.Y.); try101_80@163.com (Y.W.); hoat77@sina.com (C.-T.Z.)

**Keywords:** Homer1b/c, amyotrophic lateral sclerosis, SOD1 G93A, lithium, valproic acid (VPA)

## Abstract

Background: Mutations in the Cu/Zn superoxide dismutase (*SOD1*) gene have been linked to amyotrophic lateral sclerosis (ALS). However, the molecular mechanisms have not been elucidated yet. Homer family protein Homer1b/c is expressed widely in the central nervous system and plays important roles in neurological diseases. In this study, we explored whether Homer1b/c was involved in SOD1 mutation-linked ALS. Results: In vitro studies showed that the SOD1 G93A mutation induced an increase of Homer1b/c expression at both the mRNA and protein levels in NSC34 cells. Knockdown of Homer1b/c expression using its short interfering RNA (siRNA) (si-Homer1) protected SOD1 G93A NSC34 cells from apoptosis. The expressions of Homer1b/c and apoptosis-related protein Bax were also suppressed, while Bcl-2 was increased by lithium and valproic acid (VPA) in SOD1 G93A NSC34 cells. In vivo, both the mRNA and protein levels of Homer1b/c were increased significantly in the lumbar spinal cord in SOD1 G93A transgenic mice compared with wild type (WT) mice. Moreover, lithium and VPA treatment suppressed the expression of Homer1b/c in SOD1 G93A mice. Conclusion: The suppression of SOD1 G93A mutation-induced Homer1b/c upregulation protected ALS against neuronal apoptosis, which is a novel mechanism of the neuroprotective effect of lithium and VPA. This study provides new insights into pathogenesis and treatment of ALS.

## 1. Introduction

Amyotrophic lateral sclerosis (ALS) is a fatal neurodegenerative disease characterized by progressive muscle atrophy and four-limb paralysis due to the loss of both upper and lower motor neurons in the cortex, brainstem, and spinal cord [1]. Familial ALS (fALS), which accounts for 10% of total ALS cases, is commonly caused by mutations in a heterogeneous set of genes. To date, mutations in 18 genes and loci have been implicated in fALS. The mutations in the first identified mutant gene, the Cu/Zn superoxide dismutase (*SOD1*), are present in about 20% of fALS cases [2]. SOD1 mutation-induced degeneration of motor neurons involves mitochondrial dysfunction, increased oxidative stress, overactivation of glutamate receptors and glutamatergic neurotoxicity, SOD1 mutation-mediated neurotoxicity, intracellular calcium overload, abnormal axonal transport, excessive autophagy, and endoplasmic reticulum stress [3,4,5,6]. A large body of evidence has uncovered that apoptosis plays important roles in motor neuron degeneration produced by mutant SOD1 (mtSOD1) in ALS. The proapoptotic protein Bax was found to be significantly increased, while antiapoptotic protein Bcl-2 decreased significantly in spinal cord motor neurons of ALS patients and mtSOD1 (G93A) transgenic mice [7,8]. In agreement, it was reported that administration of *N*-benzyloxycarbonyl-Val-Asp-fluoromethyl ketone (zVAD-fmk), a caspase inhibitor, was able to inhibit neuronal apoptosis and delay the onset and mortality of ALS in mtSOD1 (G93A) transgenic mice, which reveals the involvement of neuronal apoptosis in ALS [9]. Similarly, overexpression of Bcl-2 or deletion of Bax and Bak delayed onset and halted neuronal loss, and extended survival of mice with ALS [10,11]. However, the mechanisms of fALS due to *SOD1* gene mutations have not been elucidated completely yet.

Homer1, a member of the Homer family, is expressed widely in the nervous system and consists of various isoforms. The long Homer isoforms 1b and 1c (Homer1b/c) contain an enabled/vasodilator-stimulated phosphoprotein homology 1 (EVH1) domain at the amino-terminal, which connects with proline-rich regions of target proteins, and a carboxy-terminal domain including a coiled-coil structure and leucine zipper motif, which participates in multimerization of long Homer proteins [12,13]. Homer1b/c protein is expressed at low levels in peripheral tissues such as skeletal and cardiac muscle, kidney, ovary, and testis [13,14,15]. Within the cells, Homer1b/c protein is mainly found where there are abundant postsynaptic density (PSD) proteins or postsynaptic membrane proteins [16]. Homer1b/c constitutively expresses and has dimerization capacity, which causes signal transduction or crosstalk between target proteins in neurons. It acts as an important regulator of neurological, physiological, and pathological processes such as maintaining dendritic spine structure and synaptic function [17,18], regulating the activity and cell-surface clustering of metabotropic glutamate receptor (mGluR)1a/5 [15], mediating an important cellular mechanism that regulates metabotropic glutamate signaling [19], regulating intracellular Ca^2+^ homeostasis [20], affecting mGluR1a/5-dependent synapse-to-nucleus communication and participating in glutamate-mediated excitotoxicity via endoplasmic reticulum and mitochondria pathways [21]. However, the role of Homer1b/c in ALS remains unknown.

Lithium and valproic acid (VPA) have been primarily used to treat psychiatric disorders for decades. Recently, there is increasing evidence that lithium and VPA produce neuroprotective effects in Alzheimer disease (AD), Parkinson’s disease (PD), Huntington’s disease (HD), ALS, and cancers [22,23]. De Bartolomeis et al. had reported that the expression of Homer1b/c was decreased significantly by chronic administration of therapeutically relevant doses of lithium and VPA in rat brain [24]. However, the therapeutic mechanisms of lithium and VPA in ALS remain unclear.

In this study, we examined the changes of Homer1b/c expression in mtSOD1 (G93A) NSC34 cell and mtSOD1 (G93A) transgenic mice, and explored the role of Homer1b/c in the pathogenesis of ALS. Furthermore, we investigated the effects of lithium and VPA on Homer1b/c expression in both in vitro and in vivo models of ALS.

## 2. Results

### 2.1. Human mtSOD1 and Wild Type (WT) SOD1 Expressions Were Detected in Amyotrophic Lateral Sclerosis (ALS) Cell Model

**Figure 1:**
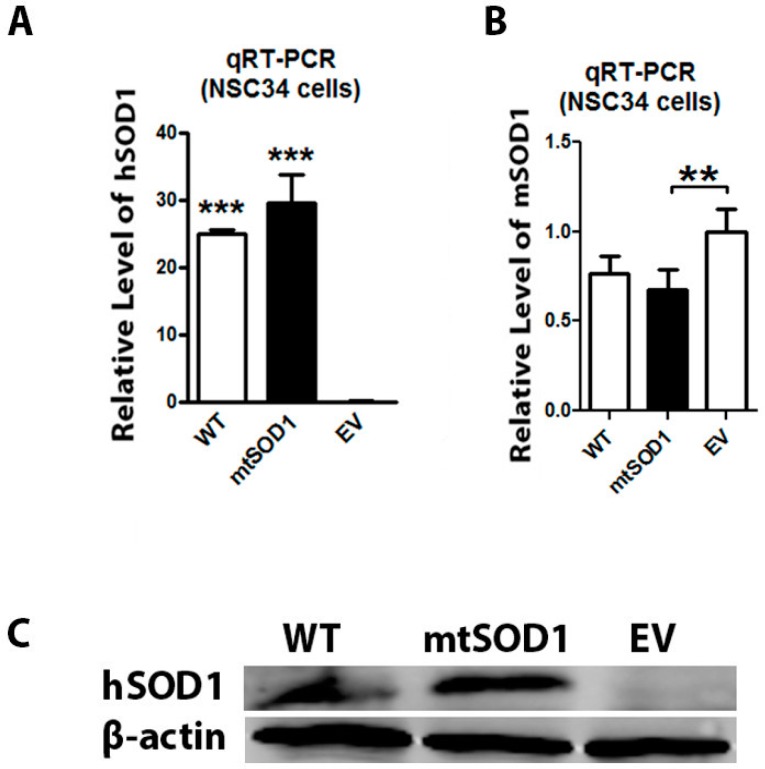
mRNA and protein expression of human SOD1 and mouse SOD1 in NSC34 cells. (**A**) The mRNA expression of human SOD1 (hSOD1) was detected by qRT-PCR in wild type (WT) SOD1 NSC34 cells and mutant SOD1 (mtSOD1) NSC34 cells, but was not detectable in empty vector (EV) NSC34 cells; (**B**) The mRNA level of mouse SOD1 (mSOD1) was detected by qRT-PCR in WT SOD1 NSC34 cells, mtSOD1 NSC34 cells, and EV NSC34 cells; (**C**) hSOD1 protein expression was measured by Western blot in WT SOD1 NSC34 cells, mtSOD1 NSC34 cells, and EV NSC34 cells. ** *p* < 0.01 vs. EV group, *** *p* < 0.001 vs. EV group. *n* = 3 independent batches of cells for each group.

NSC34 cells were stably transfected with mutant human SOD1 G93A, wild type (WT) human SOD1, and empty vector (EV) separately. We have used qRT-PCR to characterize the mRNA expression of human SOD1 (hSOD1) and mouse SOD1 (mSOD1) in the NSC34 cell line. We found that hSOD1 mRNA was expressed in both mtSOD1 NSC34 cells and WT SOD1 NSC34 cells (Figure 1A), and mSOD1 mRNA was detected in all three conditions (Figure 1B). We also used Western blot to detected the expressions of human SOD1 (mutant or WT) in the NSC34 cell line. Western blot assay showed that the human SOD1 protein expressed strongly in both mtSOD1 NSC34 cells and WT SOD1 NSC34 cells, while human SOD1 protein expression was not detectable in EV NSC34 cells (Figure 1C). These results show that exogenous human SOD1 protein was stably expressed in NCS34 cells.

### 2.2. Homer1b/c Expression Was Increased in mtSOD1 NSC34 Cells

**Figure 2:**
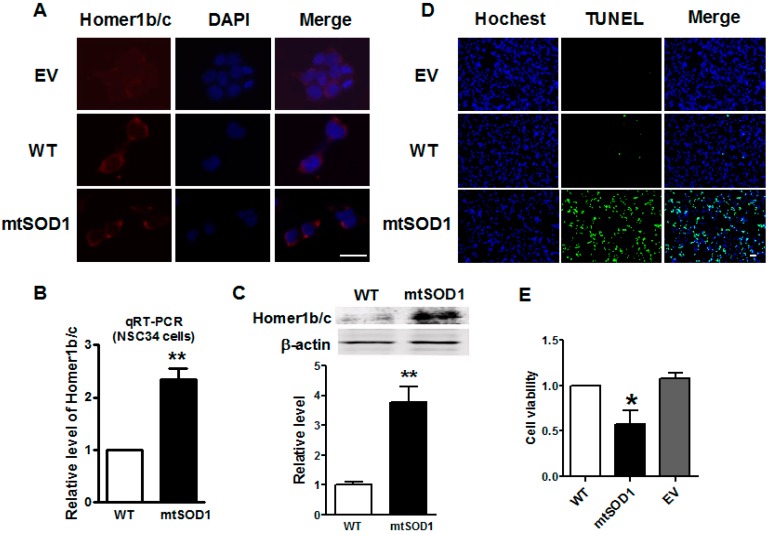
Homer1b/c expression increased in mtSOD1 NSC34 cells. (**A**) Homer1b/c protein was detected by immunofluorescence assay in cytoplasm in both wild type (WT) SOD1 NSC34 cells and SOD1 G93A (mtSOD1) NSC34 cells. Scale bar is 25 µm; (**B**) qRT-PCR detected the mRNA expression of Homer1b/c in WT SOD1 NSC34 and mtSOD1 NSC34 cells; (**C**) Homer1b/c protein expression level was measured by Western blot in WT SOD1 NSC34 cells and mtSOD1 NSC34 cells; (**D**) TUNEL staining of NSC34 cells transfected with empty vector (EV), WT SOD1, and mtSOD1. Scale bar is 20 µm; (**E**) MTT assay of NSC34 cells transfected with empty vector (EV), WT SOD1, and mtSOD1. * *p* < 0.05 vs. WT group, ** *p* < 0.01 vs. WT group. *n* = 3 independent batches of cells for each group.

Immunofluorescence assay showed that Homer1b/c protein was located in the cytoplasm of NSC34 cells and increased significantly in mtSOD1 NSC34 cells compared with WT SOD1 NSC34 cells (Figure 2A). Figure 2B shows that the mRNA level of Homer1b/c was significantly increased in mtSOD1 NSC34 cells as well. Western blot assay identified that the protein level of Homer1b/c was significantly increased in mtSOD1 NSC34 cells compared with WT SOD1 NSC34 cells (Figure 2C). This indicates that mutant human SOD1 G93A in NSC34 cells induces Homer1b/c protein overexpression. To evaluate the effects of mutant human SOD1 G93A on NSC34 cells, we investigated apoptosis by TdT-mediated dUTP nick end labeling (TUNEL) staining and detected the cell viability by 3-(4,5-dimethylthiazol-2-yl)-2,5-diphenyltetrazolium bromide (MTT) assay (Figure 2D,E). The results showed that mutant SOD1 in NSC34 cells caused cell apoptosis and the decrease of cell viability.

### 2.3. Knockdown of Homer1b/c Protected mtSOD1 NSC34 Cells from Apoptosis

**Figure 3:**
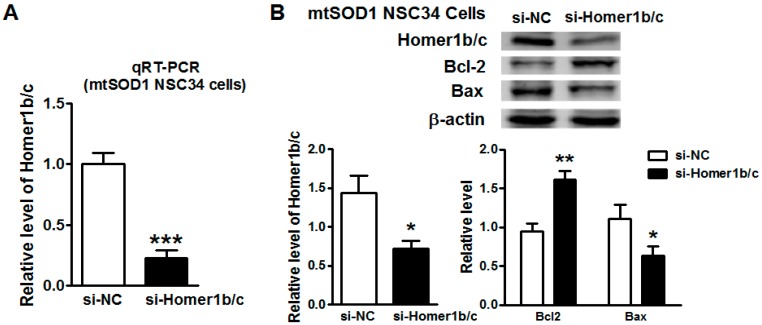
Knockdown of Homer1b/c protected mtSOD1 NSC34 cells from apoptosis. (**A**) The mRNA level of Homer1b/c was detected by qRT-PCR in mtSOD1 NSC34 cells after treatment with short interfering RNA (siRNA) negative control (NC) (si-NC)/siRNA Homer1b/c (si-Homer1b/c) for 72 h; (**B**) Western blot showed the expression of Homer1b/c protein and apoptosis-related proteins (Bcl-2 and Bax) in mtSOD1 NSC34 cells. * *p* < 0.05 vs. si-NC group, ** *p* < 0.01 vs. si-NC group, *** *p* < 0.001 vs. si-NC group. *n* = 3 independent batches of cells for each group.

To assess the role of Homer1b/c overexpression in mtSOD1 NSC34 cells, we performed an RNA transient interference assay. After transfection with Homer1 small interference RNA (si-Homer1) in mtSOD1 NSC34 cells for 72 h, both the mRNA and protein expression of Homer1b/c was suppressed significantly (Figure 3A,B). Furthermore, apoptosis-related protein Bcl-2 increased significantly, while Bax was decreased significantly in mtSOD1 NSC34 cells treated with si-Homer1b/c compared with cells treated with si-NC (negative control) (Figure 3B). This suggests that downregulation of Homer1b/c protects mtSOD1 NSC34 cells from apoptosis.

### 2.4. Lithium and VPA Reduced Homer1b/c and Apoptosis-Related Gene Expressions in mtSOD1 NSC34 Cells

**Figure 4:**
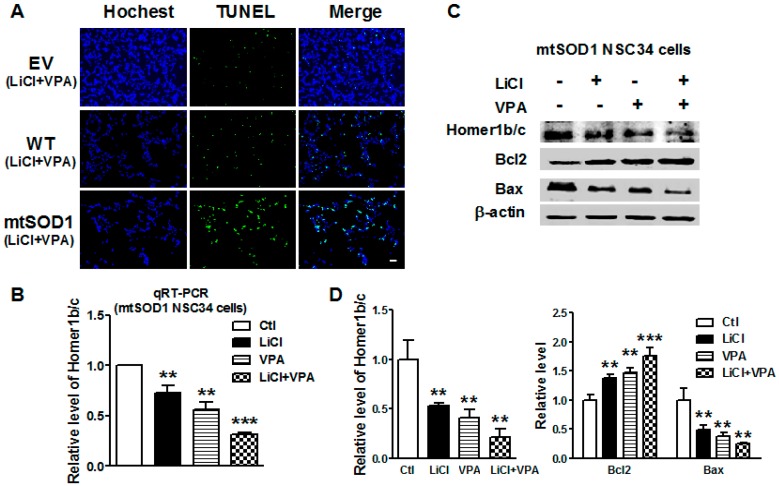
Lithium and valproic acid (VPA) reduced Homer1b/c expression and apoptosis-related genes expression in mtSOD1 NSC34 cells. (**A**) TUNEL staining of NSC34 (EV, WT SOD1, and mtSOD1) cells after treatment with LiCl + VPA (LiCl 1.0 mM + VPA 0.6 mM) for 72 h. Scale bar is 20 µm; (**B**) The mRNA level of Homer1b/c was detected by qRT-PCR in mtSOD1 NSC34 cells after treatment with LiCl (1.0 mM), VPA (0.6 mM), and LiCl + VPA (LiCl 1.0 mM + VPA 0.6 mM) for 72 h; (**C**,**D**) Western blot detected the protein levels of Homer1b/c and apoptosis- related genes (Bcl-2, Bax) expression in mtSOD1 NSC34 cells after treatment with LiCl (1.0 mM), VPA (0.6 mM), and LiCl + VPA (LiCl 1.0 mM + VPA 0.6 mM) for 72 h, + with treatment of the drug, - without treatment of the drug. ** *p* < 0.01 vs. control (Ctl) group, *** *p* < 0.001 vs. Ctl group. *n* = 3 independent batches of cells for each group.

To further verify whether lithium and VPA can regulate the expression of Homer1b/c in ALS, NSC34 (EV, WT, and mtSOD1) cells were treated with LiCl (1.0 mM), VPA (0.6 mM), and LiCl + VPA (LiCl 1.0 mM + VPA 0.6 mM) for 72 h. We firstly investigated the effects of LiCl and VPA on apoptosis of three groups NSC34 cells (Figure 4A). The results indicated that LiCl and VPA did not significantly induce cell apoptosis of normal NSC34 cells and WT SOD1 NSC34 cells. In addition, LiCl and VPA reduced the apoptosis of mtSOD1 NSC34 cells significantly compared with mtSOD1 NSC34 cells without any treatment. Compared with the control group, Homer1b/c mRNA expression was reduced significantly in mtSOD1 NSC34 cells after treatment with LiCl (*p* < 0.01), VPA (*p* < 0.01), and LiCl + VPA (*p* < 0.001) (Figure 4B). The protein level of Homer1b/c decreased after LiCl, VPA, and LiCl + VPA treatment in mtSOD1 NSC34 cells (*p* < 0.01) (Figure 4C,D). Furthermore, apoptosis-related protein Bcl-2 significantly increased after treatment with LiCl (*p* < 0.01), VPA (*p* < 0.01), and LiCl + VPA (*p* < 0.001) in mtSOD1 NSC34 cells (Figure 4C,D), while Bax protein expression decreased in mtSOD1 NSC34 cells after treatment with LiCl, VPA, and LiCl + VPA (*p* < 0.01) (Figure 4C,D). These results indicate that lithium and VPA treatment protects neurons from apoptosis by suppressing the expression of Homer1b/c in ALS.

### 2.5. Expression of Homer1b/c Was Upregulated in SOD1 G93A Transgenic Mice

**Figure 5:**
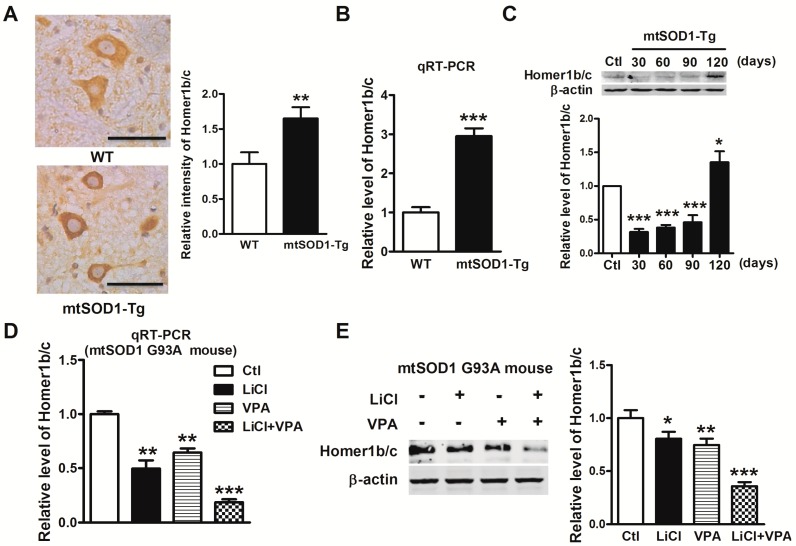
Lithium and VPA suppressed the expression of Homer1b/c in SOD1 G93A transgenic mice. (**A**) Homer1b/c protein positive neurons in the ventral horn of the lumbar spinal cords in both wild type (WT) mice and SOD1 G93A transgenic (mtSOD1-Tg) mice. Homer1b/c protein expression in neurons was detected by immunohistochemistry assay (**left**). Scale bar is 50 µm. The intensity of Hormer1b/c protein expression in neurons was analyzed by Image-Pro Plus software (Media Cybernetics, Silver Spring, MD, USA) (**right**); (**B**) The mRNA level of Homer1b/c was measured by qRT-PCR of lumbar spinal cords in both WT mice and mtSOD1-Tg mice; (**C**) Western blot detected Homer1b/c protein expression of lumbar spinal cords in WT mice and 30-, 60-, 90-, and 120-day-old mtSOD1-Tg mice; (**D**) qRT-PCR detected the mRNA level of Homer1b/c after treatment with LiCl (intraperitoneal (i.p.) 60 mg/kg), VPA (i.p. 300 mg/kg), and LiCl + VPA (i.p. LiCl 60 mg/kg + VPA 300 mg/kg) for 100 days in mtSOD1-Tg mice; (**E**) Homer1b/c protein expression was analyzed by Western blot in mtSOD1-Tg mice treated with LiCl, VPA, and LiCl + VPA for 100 days, + with treatment of the drug, - without treatment of the drug. * *p* < 0.05 vs. Ctl group, ** *p* < 0.01 vs. WT group or Ctl group, *** *p* < 0.001 vs. WT group or Ctl group. *n* = 5 independent samples for each group.

SOD1 G93A transgenic (mtSOD1-Tg) mice with overexpression of mutant human SOD1 G93A acted as an ALS mouse model in this study while, wild type human SOD1 (WT) was used as a control. Immunohistochemistry staining was used to determine the expression of Homer1b/c in lumbar spinal cords. As shown in Figure 5A, we found that Homer1b/c protein expressed evidently in neurons in the ventral horn of the lumbar spinal cords, and the expression of Homer1b/c was increased significantly in the spinal cords of mtSOD1-Tg mice compared with WT mice. Consistently, qRT-PCR and Western blot showed that both the mRNA and the protein levels of Homer1b/c were increased (Figure 5B). To investigate the time course of Homer1b/c protein expression in mtSOD1 transgenic mice, we detected the Homer1b/c protein level in lumbar spinal cords at 30-, 60-, 90-, and 120-day-old mice by Western blot assay (Figure 5C). Homer1b/c was upregulated significantly in the spinal cords of 120 days mtSOD1-Tg mice. These results suggest that Homer1b/c is involved in the pathophysiological process of ALS.

### 2.6. Lithium and VPA Suppressed the Expression of Homer1b/c in SOD1 G93A Transgenic Mice 

We further investigated the effect of lithium and VPA on the expression of Homer1b/c in the mouse model of ALS. We treated mtSOD1-Tg mice with LiCl (60 mg/kg), VPA (300 mg/kg), or LiCl (60 mg/kg) + VPA (300 mg/kg) twice daily for 100 days. The mRNA level of Homer1b/c decreased significantly in LiCl group (*p* < 0.01), VPA group (*p* < 0.01), and LiCl + VPA group (*p* < 0.001) compared to control group (Figure 5D). Consistently, after treatment with LiCl and VPA, Homer1b/c protein expression was suppressed significantly in the LiCl group (*p* < 0.05), VPA group (*p* < 0.01), and LiCl + VPA group (p < 0.001) compared with control group as well (Figure 5E). This suggests that lithium and VPA alleviate ALS partially through suppressing Homer1b/c.

## 3. Discussion

In the present study, we found that Homer1b/c was significantly overexpressed in mtSOD1 (G93A) NSC34 cells and mtSOD1 (G93A) mice. Knockdown of Homer1b/c suppresses mtSOD1 (G93A)-induced neuronal apoptosis. Lithium and VPA synergistically reduced the expression of Homer1b/c in mtSOD1 (G93A) NSC34 cells and mtSOD1 (G93A) mice. This study provides the first detailed characterization of Homer1b/c in the pathogenesis of ALS and also uncovers a new mechanism of the neuroprotective effect of lithium and VPA in ALS.

In mammals, Homer1 is expressed widely in the nervous system. Numerous studies detected Homer1 protein in brain, the dorsal horn of the spinal cord, and dorsal root ganglion and retinal ganglion cells, but Homer1 expression in the ventral horn of the spinal cord is still rarely reported. Yao et al. reported that in the dorsal horn of rats there was a high level of Homer1b/c expression, while in the ventral horn the expression of Homer1b/c was almost undetectable [25]. However, Tappe et al. found that Homer1b/c was strongly expressed throughout the spinal cord of rats, both in the dorsal horn and in the ventral horn, using immunohistochemistry [16]. The discrepancies may be due to the differences in research subjects and/or the sensitivity of different test methods and reagents used in the studies.

In this study, we found that Homer1b/c expression significantly increased at both mRNA and protein levels in an ALS cell model. We also verified this result in the spinal cord of a mouse model of ALS. The Homer1b/c was expressed both in the dorsal horn and in the ventral horn of the spinal cords in mtSOD1 (G93A) transgenic mice and WT mice. Our findings were consistent with that of Tappe et al. [16]. The level of Homer1b/c expression increased in mtSOD1 (G93A) transgenic mice compared with WT mice. Both in vitro and in vivo studies confirmed that mtSOD1 induced Homer1b/c overexpression.

We then further assessed the role of Homer1b/c overexpression in the ALS cell model. A previous study has reported that neuronal apoptosis is associated with the onset and mortality of ALS [9]. In spinal cord motor neurons, Bax protein was increased and Bcl-2 was significantly decreased in ALS patients [7]. Thus, we detected the expression of two apoptotic-related genes, Bcl-2 and Bax, in mtSOD1 (G93A) NSC34 cells. Interestingly, knockdown of Homer1b/c significantly increased Bcl-2 and decreased Bax expression. Cui et al. reported that upregulation of Homer1b/c was related to the subsequent apoptosis and downregulation of Homer1b/c, and decreased the proportion of Bax/Bcl-2 in neurons during neuroinflammation [17]. It suggested that Homer1b/c induced neuronal apoptosis through the Bax/Bcl-2 pathway in ALS, and knockdown of Homer1b/c partially suppressed mtSOD1 (G93A)-induced neuronal apoptosis. Overexpression of Homer1b/c will induce cell damage via intracellular calcium overload, glutamate-mediated excitotoxicity, and calcium-dependent production of reactive oxygen species (ROS). Thus, the inhibition of Homer1b/c protected ALS neurons not only via the apoptosis pathway, but also by other pathways, which need further study.

Lithium and VPA are two common mood-stabilizing drugs and are used to treat bipolar disorder [26]. It was reported that lithium and VPA had neuroprotective effects in vivo and in vitro via inhibition of the glycogen synthase kinase-3 (GSK-3) pathway [27]. Lithium modifies pathological cascades implicated in many neurodegenerative disorders, which includes Alzheimer’s disease (AD), Huntington’s disease (HD), multiple system atrophy (MSA), and ALS [28]. However, lithium treatment for ALS patients is not the best strategy in clinical trials. The effects of lithium on ALS are mainly dependent on the beneficial therapeutic window for lithium treatment, the blood lithium levels, and route of administration and frequency [29]. It has recently been shown that neuroprotection by lithium is antagonized by riluzole (the only FDA-approved drug for ALS), suggesting that the drug’s neurotoxic effects may cover up the potential neuroprotective action of lithium [30]. The beneficial effect of VPA has been documented in different neurodegenerative experimental models, including ALS [31]. Our previous studies showed that lithium and VPA delayed the disease symptom onset, increased survival time, reduced neurological deficits in mtSOD1 (G93A) mice, and that the combination of lithium and VPA was more consistent than lithium or VPA alone [32]. Recently, a 21-month clinical trial showed that cotreatment with lithium and VPA significantly increased survival rate and exerted a neuroprotective effect in patients with sporadic ALS [33]. In our study, the effects of LiCl and VPA cotreatment on the ALS model are better than with single LiCl and VPA separately. Therefore, we believe that there is a need for better-designed preclinical studies to present the potential beneficial effects of lithium for ALS. Our recent research showed that lithium and VPA inhibited the activation of the Notch pathway in mtSOD1 (G93A) mice [34]. However, the effects of lithium and VPA on Homer1b/c expression were unknown. In vitro study showed that Homer1b/c mRNA expression was significantly reduced after treatment with LiCl and VPA. Furthermore, apoptosis-related protein Bcl-2 increased significantly, while Bax expression decreased after treatment with LiCl and VPA. This suggests that lithium and VPA treatment protect neurons from apoptosis through suppressed expression of Homer1b/c. We also found that lithium and VPA significantly decreased the expression of Homer1b/c in mtSOD1 (G93A) mice. In particular, combined treatment of LiCl and VPA had synergistic effects on the expression of Homer1b/c. This suggests that lithium and VPA synergistically alleviate ALS by suppressing the expression of Homer1b/c, and downregulation of Homer1b/c is a new potential mechanism of the neuroprotective effect of lithium and valproic acid.

## 4. Materials and Methods

### 4.1. Cell Culture and Treatment

The plasmid of human SOD1 gene with G93A mutation is stably transfected into NSC34 cell line (Cedarlane Laboratories, Vancouver, BC, Canada), a hybrid cell line of mouse neuroblastoma and embryonic spinal motor neurons, named as SOD1 G93A (mtSOD1) NSC34 cell line. In this study, the mtSOD1 NSC34 cell line acted as a cell model of ALS, while NSC34 cell line transfected with wild type human SOD1 (WT) was used as control. The cells were cultivated in Dulbecco’s Modified Eagle’s Medium-High Glucose (GE Healthcare Life Sciences Hyclone, Pittsburgh, PA, USA) with 10% fetal bovine serum (FBS), 100 U/mL penicillin/streptomycin (GE Healthcare Life Sciences Hyclone), and 200 µg/mL of puromycin (G418, Life Technologies Corporation, Grand Island, NY, USA), which was selected to maintain the stable cell line translation. Cells were grown in a humidified atmosphere of 5% CO_2_ in air at 37 °C. mtSOD1 NSC34 cells were treated with LiCl (Sigma, St. Louis, MO, USA) 1.0 mM, VPA (Sigma) 0.6 mM, and LiCl plus VPA (LiCl 1.0 mM + VPA 0.6 mM), with 0.9% normal saline as control. After treatment for 72 h, mtSOD1 NSC34 cells were collected for further total RNA and protein extraction.

### 4.2. Animals and Drug Treatments

Transgenic mice with a mutant *SOD1 G93A* gene (B6SJL-Tg (SOD1-G93A) 1Gur/J) overexpression were used as a mouse model of ALS, which were obtained from Jackson Laboratory (Bar Harbor, ME, USA). The animal protocol was approved by the Institutional Animal Care and Use Committee at Harbin Medical University (No. HMUIRB-2008-06) and the Institute of Laboratory Animal Science of China (A5655-01). Mice were administered with LiCl (60 mg/kg), VPA (300 mg/kg), or LiCl (60 mg/kg) plus VPA (300 mg/kg) twice daily from the 30th day after birth by intraperitoneal (i.p.) injection for 100 days. The mice in control group were treated with the same volume of saline.

### 4.3. Quantitative Reverse Transcription Polymerase Chain Reaction (qRT-PCR)

Total RNA was extracted from NSC34 cells and mouse spinal cords using TRIzol (Life Technologies) according to the manufacturer’s instructions. β-Actin was used as the internal control. The primer pairs are as follows: hSOD1 (NM_000454.4) Forward: 5′-TGGGCCAAAGGATGAAGAG-3′, Reverse: 5′-TTACACCACAAGCCAAACGAC-3′; mSOD1 (NM_011434.1) Forward: 5′-GTCCGTCGGCTTCTCGTC-3′, Reverse: 5′-ACCGCTTGCCTTCTGCTC-3′; Homer1 (NM_001284189) Forward: 5′-CTGAACCAGACAGTGCAGGA-3′, Reverse: 5′-TACTGCGGAAAGCCTCTTGT-3′; β-actin (NM_007393.5) Forward: 5′-CCAGCCTTCCTTCTTGGGTAT-3′, Reverse: 5′-TGCTGGAAGGTGGACAGTGAG-3′. We determined the appropriate cycle threshold (*C*_t_) using the automatic baseline determination feature and analyzed data by relative quantitative analysis method.

### 4.4. Western Blot

NSC34 cells and mouse spinal cords were homogenized in RIPA lysis buffer (Thermo Scientific, Grand Island, NY, USA) and extract was centrifuged for 10 min at 14,000× *g* in 4 °C centrifuge. Total protein samples (30–100 µg) were analyzed by 10% sodium dodecyl sulfate polyacrylamide gel electrophoresis (SDS-PAGE) gel and transferred to polyvinylidene difluoride (PVDF) membranes by a wet blotting procedure (100 V, 120 min, 4 °C). Blocking buffer (5%) incubation was followed by incubation with primary antibodies at 4 °C overnight using the following concentration: SOD1 (ab52950; Abcam, Cambridge, MA, USA) 1:1000, Homer1b/c (sc-25271, Santa Cruz, Dallas, TX, USA) 1:1000, Bcl-2 (Zhongshan Jinqiao Biotechnology, Beijing, China) 1:1000, Bax (Zhongshan Jinqiao Biotechnology) 1:1000, and β-actin (Santa cruz Biotechnology, Dallas, TX, USA) 1:1000, which acted as the internal control for normalization of protein expression. Primary antibody incubation was followed by alkaline phosphatase-conjugated secondary antibody. Visualization of bound antibody was achieved with film (Kodak, Rochester, NY, USA) by using 5-bromo-4-chloro-3-indolyl phosphate (BCIP)/nitro blue tetrazolium (NBT) (Beyotime Institute of Biotechnology, Jiangsu, China).

### 4.5. Transient Interference Assay

Homer1 small interference RNA (si-Homer1) was used to silence the expression of Homer1b/c in SOD1 G93A NSC34 cells. The final concentration of 100 nM si-Homer1 (sc-35582, Santa Cruz Biotechnology) or the negative control siRNA (si-Control, sc-37007, Santa Cruz Biotechnology) were delivered by Lipofectamine™ RNAiMAX (Life Technologies) in 6-well plates and incubated for 72 h at 37 °C in a CO_2_ incubator. All the transfections were repeated more than three times independently.

### 4.6. Immunohistochemical Staining

Paraformaldehyde (4%)-fixed and paraffin-embedded tissues from the spinal cords of mouse were used for immunohistochemical staining. Six-micrometer sections were cut from paraffin-embedded spinal cord tissues and deparaffinized. Then, the sections were blocked with 10% nonimmune goat serum (Zhongshan Jinqiao Biotechnology) and incubated overnight with primary anti-Homer1b/c antibody (sc-25271, Santa Cruz) (1:200) at 4 °C. The signals were detected with DAB (3,3′-diaminobenzidine) substrate (Zhongshan Jinqiao Biotechnology). Hematoxylin was used as a counterstain. The sections were observed by Leica microscope (Leica, Wetzlar, Germany). The immunohistochemical staining images were analyzed by Image-Pro Plus 6.0 software (Media Cybermetics, Inc., Silver Spring, MD, USA).

### 4.7. MTT Assay

The number of viable cells was estimated using the MTT assay. NSC34 cells (4 ×10^4^ cells/well) were plated into 96-well plates and cultured at 37 °C for 24 h. After 24h incubation, the medium was removed from each well and 100 µL of the culture medium comprising 20 µL of MTT reagent (5 mg/mL) was added into wells and incubated for 4 h at 37 °C. After this incubation period, 100 µL of dimethyl sulfoxide was added, and the optical density of each well was measured at 570 nm using an ELISA plate reader (BioTek Instruments, Inc., Winooski, VT, USA).

### 4.8. TUNEL Staining

NSC34 (EV, WT, and mtSOD1) cells were seeded into 6-well plates and cultured at 37 °C for 24 h, then Hoechst-stained. Apoptotic NSC34 cells were detected with TUNEL using a commercial kit (Roche, Basel, Switzerland) according to the manufacturer’s protocol and observed using fluorescence microscopy (Olympus, Tokyo, Japan).

### 4.9. Statistical Analysis

Student’s *t*-test was used to compare the difference between two groups. Statistical comparisons among multiple groups were performed by Tukey’s *post hoc* test and one-way ANOVA. All data are presented as mean ± SEM, and *p* < 0.05 was considered as significantly different.

## 5. Conclusions

Our study showed that Homer1b/c increased in mtSOD1 G93A transgenic mice, which contributes to neuronal apoptosis in ALS. The inhibition of Homer1b/c by its siRNA and lithium and VPA protected against neuronal apoptosis in ALS induced by mtSOD1 G93A. Our findings suggest that Homer1b/c is a new potential regulator or drug target in the treatment of ALS.
